# Ultra-strong long-chain polyamide elastomers with programmable supramolecular interactions and oriented crystalline microstructures

**DOI:** 10.1038/s41467-019-09218-6

**Published:** 2019-03-21

**Authors:** Lingzhi Song, Tianyu Zhu, Liang Yuan, Jiangjun Zhou, Yaqiong Zhang, Zhongkai Wang, Chuanbing Tang

**Affiliations:** 1 Biomass Molecular Engineering Center, Anhui Agricultural University, Hefei, Anhui 230036 China; 2 Department of Chemistry and Biochemistry, University of South Carolina, Columbia, SC 29208 USA

## Abstract

Polyamides are one of the most important polymers. Long-chain aliphatic polyamides could bridge the gap between traditional polyamides and polyethylenes. Here we report an approach to preparing sustainable ultra-strong elastomers from biomass-derived long-chain polyamides by thiol-ene addition copolymerization with diamide diene monomers. The pendant polar hydroxyl and non-polar butyrate groups between amides allow controlled programming of supramolecular hydrogen bonding and facile tuning of crystallization of polymer chains. The presence of thioether groups on the main chain can further induce metal–ligand coordination (cuprous-thioether). Unidirectional step-cycle tensile deformation has been applied to these polyamides and significantly enhances tensile strength to over 210 MPa while maintaining elasticity. Uniaxial deformation leads to a rearrangement and alignment of crystalline microstructures, which is responsible for the mechanical enhancement. These chromophore-free polyamides are observed with strong luminescence ascribed to the effect of aggregation-induced emission (AIE), originating from the formation of amide clusters with restricted molecular motions.

## Abstract (web summary)

Long-chain polyamides could bridge the gap between traditional polyamides and polyethylenes. Here the authors show the preparation of diamide diene monomers derived from natural resources coupled by thiol-ene addition copolymerization to form long-chain amide-containing polymers for the synthesis of ultra-strong elastomers.

## Introduction

Long-chain aliphatic polyesters and polyamides possess some of unique compositions and properties, given that they bridge the gap between conventional polyethylene and short-chain condensation polymers (or polycondensates)^1–4^. While the long aliphatic chain promotes crystallization via van der Waals interactions, the presence of functional groups (e.g., esters and amides) powers this class of polymers for applications that polyethylene cannot enable such as degradability and compostability^5,6^. Coupling with possible feedstocks from renewable natural resources, these type of polymers would hold enormous potential in pursuing sustainability toward green bioplastics^7–17^. Thus, there is an indigenous incentive to design viable routes to access long-chain monomers and their corresponding polymers. One of the primary approaches is to prepare linear α, ω-difunctional monomers, including selective terminal functionalization of fatty acids, which are corroborated by recent advances in catalytic conversions of plant oils^18–20^. However, many of these polymers require tedious synthetic processes and exhibit inferior physiochemical properties compared with either polyethylene or traditional condensation polymers. Most of them are reported as thermoplastics with just a few on elastomers with mediocre mechanical properties.

**Fig. 1:**
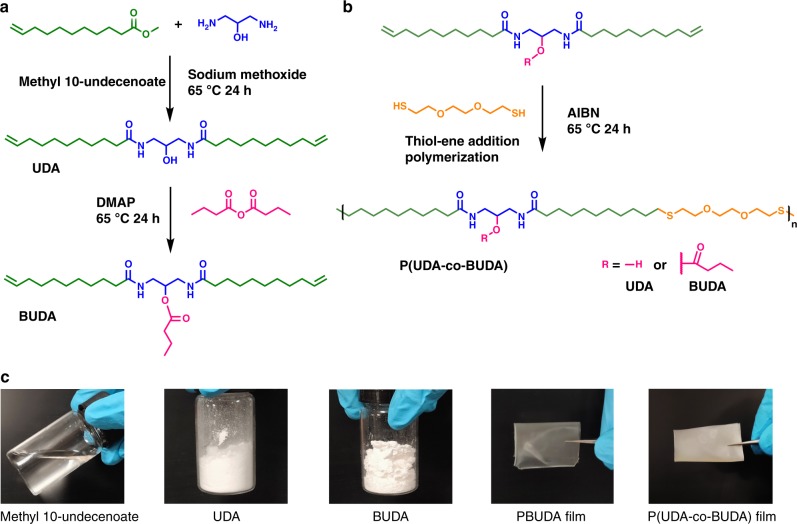
Design of monomers and functional polyamides. **a** Synthesis of amide diene monomers from a castor oil derivative; **b** synthesis of polyamides by thiol-ene addition polymerization; **c** photos represent methyl 10-undecenoate, amide diene monomers (UDA: *N,N'*-(2-hydroxypropane-1,3-diyl)bis(undec-10-enamide), BUDA: 1,3-di(undec-10-enamido)propan-2-yl butyrate), PBUDA and P(UDA-*co*-BUDA) films

As shown in Fig. 1, we conceptualized castor oil-derived amide diene monomers. The key advantage of our design is the introduction of hydroxyl groups in the main chain that could be further used to install functional groups as side chains, which has been rarely reported with other polyamide systems. Two monomers with hydroxyl or butyrate pendant groups were first synthesized. Functional polyamides were then prepared via thiol-ene addition polymerization. Copolymerization of these monomers would allow precise tuning of crystallization properties of resultant polyamides. The existence of butyrate pendant groups may restrain the formation of a highly crystalline structure, but facilitate an elastic amorphous matrix. Nanocrystals from the packing of linear alkyl chains and supramolecular hydrogen bonding from amide/hydroxyl side groups contribute to high mechanical strength. Moreover, it is worth noting that the ultrahigh mechanical strength could be achieved via the formation of oriented microstructures^21–23^. Unidirectional step-cycle tensile deformation was therefore performed on these functional polyamides to induce alignment of microstructures. Ultra-strong elastomers (uEs) with oriented nanocrystals dispersed in the amorphous matrix were obtained.

On a different perspective, a variety of small molecules and macromolecules exhibit enhanced luminescence rather than quenching at the solid state, a phenomenon coined as aggregation-induced emission (AIE) by Tang et al.^24–26^. Very interestingly, many chromophore-free synthetic polymers (e.g., anhydride, amide-rich polymers)^27–29^ and natural biopolymers (e.g., cellulose, starch, peptide)^27^ also show unusual emission at the aggregation state. Given the rich content of amide groups, we hypothesize that our polyamides could have similar amide cluster-induced luminescence, which could bring additional benefits of this class of materials.

We report herein the design of a class of biobased long-chain aliphatic polyamides that combine van der Waals interactions and supramolecular hydrogen bonding. The macromolecular compositions facilitate the formation of crystalline nanostructures dispersed in an amorphous matrix. With the controlled programming of supramolecular interactions, it would allow the facile tuning of mechanical strength. Further unidirectional stretching of these polyamide films induces the orientation of ordered crystalline domains and thus results in ultra-strong elastic materials with an increase of tensile strength by nearly one order of magnitude. This kind of biobased polyamides is the strongest elastomers reported among long-chain aliphatic polycondensates.

## Results

### Synthesis of long-chain polyamides

Recently, several approaches have been developed to prepare polyamides with functional side groups^30–33^. However, the synthesis of monomers was mostly involved with tedious work-up and relatively low yields. In this work, we developed efficient synthesis of diene monomers that bear two secondary amide bonds in the main chain attached with pendant side groups. As shown in Fig. 1a, an α, ω-diene amide monomer with hydroxyl side group (*N,N'*-(2-hydroxypropane-1,3-diyl)bis(undec-10-enamide), UDA) was first prepared via amidation of methyl 10-undecenoate with 1,3-diamino-2-propanol^34–36^. The reaction conversion into UDA monomer during this step is quantitative (~100%). The hydroxyl group in UDA was then reacted with butyric anhydride to introduce a larger pendant group that could later inhibit crystallization of its polymer. The resultant monomer 1,3-di(undec-10-enamido)propan-2-yl butyrate is labeled as BUDA. The successful synthesis of UDA and BUDA was confirmed by Fourier-transform infrared spectroscopy (FT-IR), proton nuclear magnetic resonance (^1^H NMR), and carbon-13 nuclear magnetic resonance spectroscopy (Supplementary Fig. 1). Thiol-ene addition polymerization of both diene monomers was performed to afford polyamides (Fig. 1b, labeled as PUDA and PBUDA, respectively). The existence of hydrogen bonded amide groups along with linear alkyl chains would result in highly crystalline polymers for PUDA. Indeed, PUDA homopolymer (P7) is a highly crystalline and brittle material (Supplementary Fig. 2a). On the other hand, the presence of pendant groups could interrupt the formation of crystalline domains in PBUDA (P0, transparent in Fig. 1c). Thus, we predict that a copolymer of both monomers (P(UDA-*co*-PBUDA) (opaque in Fig. 1c) could have the existence of a two-phase morphology with nanocrystalline domains dispersed in an amorphous matrix. The precise control of feed ratios of co-monomers would allow the facile tuning of crystallinity with the aid of programmable supramolecular hydrogen bonding.

To understand the effect of monomeric compositions on microstructures and thermomechanical properties of functional polyamides, we prepared a series of P(UDA-*co*-BUDA) copolymers with the fraction of UDA varying from 10 to 80 mol% (polyamides P1–P6 in Supplementary Table 1). The formation of these polyamide copolymers was confirmed by ^1^H NMR (Supplementary Fig. 3), gel permeation chromatography (Supplementary Fig. 4), and thermogravimetric analysis (Supplementary Fig. 5). All these polyamides have relatively high molecular weight (>20,000 g mol^−1^) and good thermal stability.

**Fig. 2:**
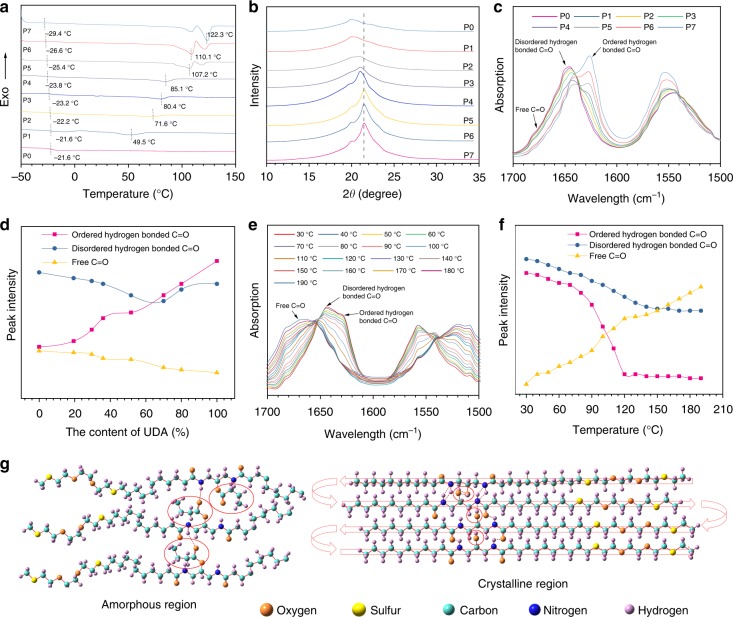
Characterization of polyamides P0–P7. **a** Differential scanning calorimetry (DSC) heating curves; **b** one-dimensional (1D) wide-angle X-ray diffraction (WAXD) profiles; **c** Fourier-transform infrared spectroscopy (FT-IR) spectra in the 1500–1700 cm^−1^ region for P0–P7; **d** the change of FT-IR peak intensity of free/disordered/ordered hydrogen bonded C=O for P0–P7 as a function of UDA content; **e** variable temperature FT-IR spectra of P6 in the 1500–1700 cm^−1^ region; **f** the change of FT-IR peak intensity of free/disordered/ordered hydrogen bonded C=O for P6 as a function of temperature; **g** proposed molecular models for crystalline domain and amorphous region

Differential scanning calorimetry (DSC) was performed to estimate the microstructure of polymers. Figure 2a shows heat flow curves of DSC for P0–P7. All copolymers exhibited glass transition temperature (*T*_g_) far below room temperature (−29.4 to −21.6 °C). Moreover, DSC curves show distinct melting and crystallization processes for both PUDA homopolymer (P7) and copolymers (P1–P6). The melting temperature (*T*_m_) of P1–P7 increased with the increase of UDA content. PUDA homopolymer (P7) has the highest *T*_m_ at ~122.3 °C and an enthalpy of fusion (Δ*H*_m_) of 595.5 J g^−1^, while PBUDA homopolymer (P0) was not observed with a melting point. The DSC heating curves for polymers with high UDA content (P5–P7) show two distinct melting peaks, which can be interpreted by a coexistence of the γ-phase and α′-phase crystals at elevated temperature^37,38^. These results suggest the incorporation of UDA with the hydroxyl side group facilitates the formation of crystalline domains: the higher the UDA content, the higher melting temperature.

### Microstructures and supramolecular hydrogen bonding

Wide-angle X-ray diffraction (WAXD) was further used to probe crystallization behaviors of homopolymers of UDA and BUDA, as well as their copolymers (Fig. 2b). Except PBUDA homopolymers, all other polymers were observed with a broad baseline and sharp peaks with varied intensity, indicating the coexistence of both crystalline and amorphous microstructures. The crystalline and amorphous peaks were deconvoluted via peak fitting (Supplementary Fig. 6). The crystalline peak at 2*θ* around 21.2° corresponds to the γ-crystalline form as reported for polyamides^39^. The degree of crystallinity (*X*_c_) was calculated based on the fraction of areas under crystalline peaks over the total areas under both crystalline and amorphous regions. Supplementary Table 2 summarizes the peak positions and *X*_c_ for P0–P7^40^. *X*_c_ increases with the increase of UDA content in the copolymers, in good agreement with the DSC data. The size of crystalline domains (*t*) can be estimated from the Scherrer’s formula: *t* = *λ*/*B* cos *θ*, where *λ* is the wavelength of X-ray, *B* is the full width at half-maximum of diffraction peaks, and *θ* is the diffraction angle. As a result, the size of crystalline domains is only ca. 6.7–8.5 nm for the copolymers. Although well-aligned UDA segments induce the growth of crystallites, the pendant butyrate groups hinder the alignment polyethylene-like chains at the backbone and further depress the overall strength of intermolecular van der Waals interactions. Supplementary Figure 7 shows a polarized optical micrograph (POM) of a representative copolymer P4, the nearly dark image also indicated that the crystals are too small to be visualized by POM.

It was conceptualized that supramolecular hydrogen bonding should play a critical role in the crystallization of polyamides. As hydrogen bonding is temperature-sensitive, these copolymers were characterized by variable temperature FT-IR spectroscopy. When C=O groups of amides form hydrogen bonds, their infrared absorption peak may shift. Figure 2c shows FT-IR spectra of P0–P7 in the 1500–1700 cm^−1^ region. The peaks near 1620–1680 cm^−1^ are assigned to the stretching of carbonyl groups. This region of carbonyl stretching contains three distinct contributions: free carbonyl groups at 1678 cm^−1^, disordered hydrogen bonded carbonyl groups at 1646 cm^−1^, and ordered hydrogen bonded carbonyl groups at 1626 cm^−1^, the latter two of which indicate the formation of hydrogen bonding in polyamides^41^. Figure 2d shows the changes of peak intensities of free/disordered hydrogen bonded/ordered hydrogen bonded carbonyl groups as a function of UDA content for polymers P0–P7 at room temperature. It can be seen that the peak intensity of ordered hydrogen bonded groups greatly increased with the increase of UDA content, demonstrating that the presence of more UDA facilitated the formation of hydrogen bonding. Figure 2e shows variable temperature FT-IR spectra of a representative copolymer P6 in the 1500–1700 cm^−1^ region, which indicates association/dissociation of hydrogen bonds under different temperature. With the increase of temperature, the absorption peaks at 1646 and 1626 cm^−1^ decreased, and a broad peak near 1678 cm^−1^ gradually increased, indicating the weakening and dissociation of hydrogen bonds. Figure 2f shows the changes of peak intensity of free/disordered hydrogen bonded/ordered hydrogen bonded C=O for P6 as a function of temperature. There is a transition occurred from 80 °C to 120 °C, which is in excellent agreement with the melting results by DSC. This suggested that the ordered hydrogen bonds exist mostly within the crystalline region. Based on these observations, molecular models for crystalline and amorphous phases were proposed in Fig. 2g. The large butyrate side groups of BUDA disrupt chain regularity and prefer to stay in the amorphous region, while hydroxyl groups facilitate crystallization. Overall, it can be concluded that in addition to intermolecular van der Waals interactions from linear alkyl chains, the formation of semicrystalline microstructures was largely facilitated by inter/intra-molecular hydrogen bonding.

**Fig. 3:**
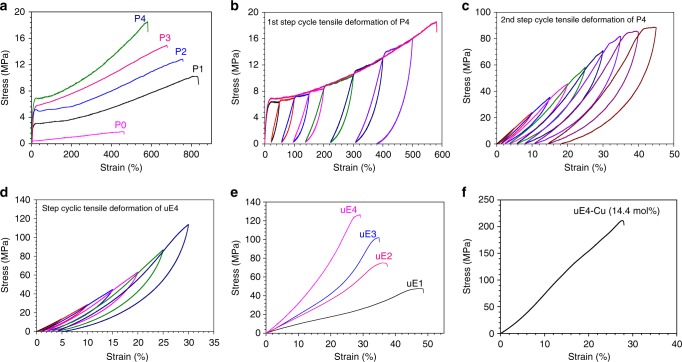
Step-cycle tensile deformation as a processing method to improve mechanical properties. **a** Monotonic stress–strain curves of P0–P4; **b** first and **c** second step-cycle tensile deformation of P4; **d** monotonic stress–strain curves of ultra-strong elastomer 4 (uE4) during step-cycle tensile deformation; **e** monotonic stress-monotonic strain curves of uE1–uE4; **f** monotonic stress–strain curve of uE4-Cu (14.4 mol%)

The dispersion of crystalline microstructures in an amorphous matrix is crucial to possess outstanding mechanical properties for many of polyamide systems^42–44^. For the copolymers of UDA and BUDA, an amorphous matrix envelopes nanocrystalline domains, where there are rich inter/intra-molecular hydrogen bonds. The mechanical properties of copolymers with various UDA contents were measured via monotonic tensile deformation (Fig. 3a, Supplementary Fig. 8, and Supplementary Table 3). Compared to the non-crystalline homopolymer PBUDA, copolymer P4 has tensile strength and Young’s modulus at 18.4 ± 2.1 and 149.6 ± 3.1 MPa, an increase of 429.6% and 521.4%, respectively. Moreover, with the increase of UDA content, the toughness increased from 5.2 (P0) to 65.0 MJ m^−3^ (P4), an increase over 12.5-folds, demonstrating that the existence of nanocrystalline domains greatly enhances toughness. Copolymers P1–P3 were also observed with similar trends at different levels of increase in mechanical properties (Fig. 3a). Copolymers P5–P6 with higher contents of UDA (60% and 80% respectively) were not subject to such comparisons, as they are quite brittle.

### Preparation of uEs

It is expected that the as-prepared film samples of copolymers by solution casting do not possess highly oriented crystalline microstructures. Consecutive cyclic tensile deformation was applied to copolymers P1–P4 to induce the alignment of microstructures^45,46^. After deformation, these samples were labeled as uE1–uE4 (Fig. 3 and Supplementary Figs. 9–11). The stress–strain curves during the first and second step-cycle tensile deformation of P4 are shown in Fig. 3b and c. The large hysteresis during each cycle is in agreement with the Mullin effect^47^. After the step-cycle tensile deformation, these copolymers were transformed into ultra-strong elastomers (uE1–uE4). For example, the stress at break of uE4 is 126.3 MPa, more than seven times of as-prepared P4 (Fig. 3e). Though the elongation at break of uE4 reduced to 30%, its elastic recovery is strikingly high at 94.5% (Fig. 3d). Similarly, the high elastic recovery was also observed for uE1, uE2, and uE3 at 96.9%, 96.7%, and 95% respectively (Supplementary Fig. 12). Together, these elastomers combined ultrahigh tensile strength and excellent elasticity. The mechanical properties of uE1–uE4 are summarized in Supplementary Table 4. The mechanical properties of uEs can be precisely tuned by controlling the content of UDA. Supplementary Figure 13 shows that a uE4 fiber with a diameter of ~135 μm can easily hold a weight of 200 g, implying the exceptional toughness.

To demonstrate that there is much room for further improving mechanical strength of these polyamides, we carried out a preliminary study by introducing metal–ligand coordination. Cuprous–thioether coordination has been widely observed in biological systems and recently used in synthetic polymers^48–51^. Our polyamides contain a reasonable fraction of thioether groups along the backbone. Thus, CuBr was used to induce cuprous–thioether coordination to strengthen the mechanical properties (Supplementary Fig. 22). After similar tensile deformation processing, as shown in Fig. 3f and Supplementary Figs. 14–15, polyamides with 14.4 mol% cuprous ions (to the sulfur atoms, labeled as uE4-Cu) exhibit stress at break at 211.2 MPa, more than 65% increase over uE4, while maintaining similar elongation at break (27.9%). This substantial enhancement is a strong indication of the robustness of current structures of polyamide systems.

To place our work in context, we have collected a variety of long-chain polyamides and polyesters reported in literature and compared their tensile strength (Supplementary Table 5). It is quite evident that the polyamides we prepared possess the highest tensile strength, even much higher than long-chain nylons (Nylon 12). While it may not be fair to compare, our polymers also have better tensile strength than short-chain nylons (e.g., polyamide 6.6).

**Fig. 4:**
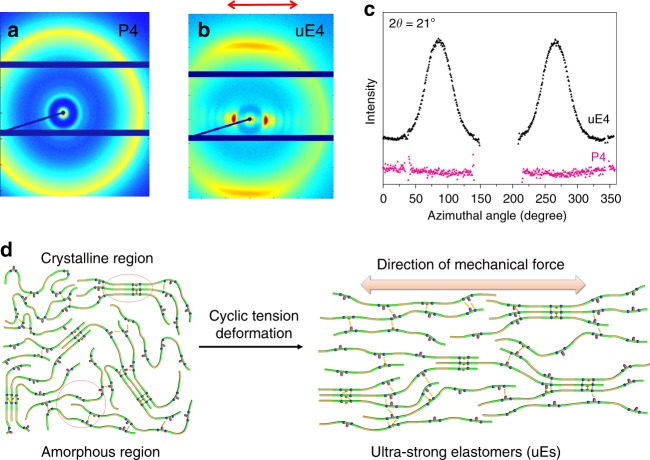
Microstructure analysis of as-prepared and tensile-deformed copolymers. **a**, **b** Two-dimensional (2D) wide-angle X-ray diffraction (WAXD) patterns of P4 and ultra-strong elastomer 4 (uE4); **c** WAXD azimuthal intensity profiles for P4 and uE4 at 2*θ* = 21°; **d** a proposed model to illustrate the microstructure rearrangement of P(UDA-*co*-BUDA) copolymers during cyclic tensile deformation

To understand the substantial difference of mechanical properties between as-prepared P1–P4 and tensile-deformed uE1–uE4, WAXD measurement was used to reveal how the microstructures could be rearranged by the cyclic tensile deformation. As shown in Fig. 4a, 2D WAXD pattern of P4 is nearly isotropic. After cyclic tensile processing, an anisotropic 2D WAXD pattern is clearly formed for uE4, the arrow represents the stretching direction (Fig. 4b). The scattering intensity was found to converge on the meridian, which indicates that crystalline domains are oriented along the tensile direction. The orientation of crystal phase is also verified by the azimuthal angle at 2*θ* = 21° (Fig. 4c). According to the WAXD analysis (Supplementary Figs. 16–18), we proposed a model on microstructure rearrangement of P(UDA-*co*-BUDA) copolymers during cyclic tensile deformation (Fig. 4d).

### AIE of polyamides

**Fig. 5:**
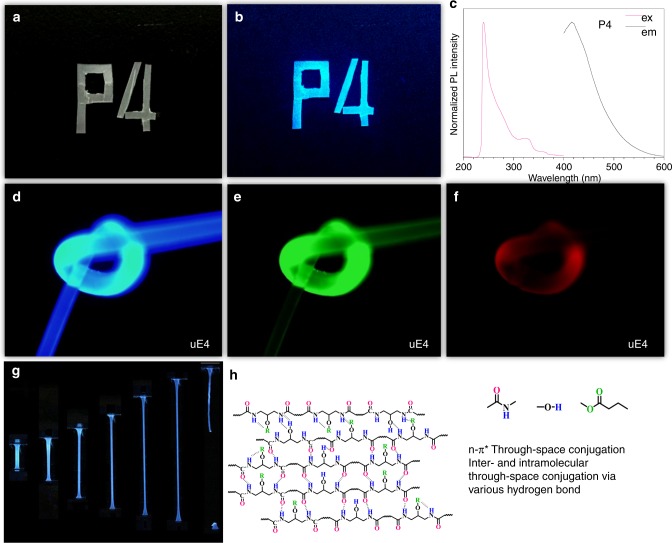
Aggregation-induced emission (AIE) properties of as-prepared polyamides and ultra-strong elastomers (uEs). **a** Photo of P4 under visible light; **b** photo of P4 under 365 nm light, emitting strong blue fluorescent; **c** ultraviolet–visible (UV–vis) absorption (red line) and fluorescent emission (black line) spectra of P4; **d**–**f** fluorescence images of uE4 fibers observed under a microscope with a diode laser under varied excited wavelengths (340–390, 460–495, and 530–550nm); **g** photos of P4 during stretching excited by 365 nm UV lamp; **h** schematic illustration on the luminescent mechanism of polyamides

Surprisingly, both as-prepared and uni-directionally stretched polyamides exhibited strong luminescence. As shown in Fig. 5a and b, as-prepared P4 emits strong blue photoluminescence. The corresponding fluorescent spectrum shows an emission peak at ~418 nm, while the UV–vis absorption peak is at 210 nm (Fig. 5c and Supplementary Fig. 19). Figure 5d–f show fluorescence images of stretched uE4 fibers observed under microscope with a diode laser, which was excited with 340–380, 460–495, and 530–550 nm, respectively. Moreover, Fig. 5g indicates that the emission color of P4 film did not show obvious difference during stretching, which was further corroborated by fluorescent spectra of P1–P4 and uE1–uE4 (Supplementary Fig. 20). Traditional chromophore-containing fluorescent polymers do not emit strong fluorescence in concentrated solutions or solid states due to the aggregation-caused quenching^24,52^. In the current case, the as-prepared polyamides without any conventional chromophores exhibit strong emission at the solid state. We believe that these polyamides have the properties of AIE (Supplementary Fig. 21), which was first discovered with chromophore-containing molecules by Tang and coworkers in 2001^25^. Recently, synthetic and natural chromophore-free polymers have been reported with AIE characteristics, as a result of the formation of polar group clusters due to restriction of molecular motions^27,53^. A few research groups have reported polyamides with photoluminescence^29,54–56^. It is generally believed that the strong hydrogen bonding induces the formation of local clusters of amides that facilitate fluorescence. We hypothesized that our polymers with rich amide and hydroxyl groups enable through-space conjugation via n–π* or π–π* transitions^52^. Thus, the as-prepared polyamides and elastomers could generate strong fluorescence in the solid state (Fig. 5h), which could be beneficial for the extension of these elastomers from bioplastics to other areas such as biomedical applications that are worthy for exploration in the future.

In summary, biobased α, ω-diene amide monomers were designed with pendant polar hydroxyl or non-polar butyrate groups. Compared with traditional polyamides, these long-chain polyamides prepared by thiol-ene addition polymerization have controlled programmable supramolecular hydrogen bonding and tunable crystallinity with mechanical properties facilely adjusted by changing co-monomer compositions. By unidirectional step-cycle tensile deformation, ultra-strong elastomers were obtained without the addition of any fillers. It is the formation of highly aligned crystalline microstructures responsible for the unique mechanical properties of elastomers. Moreover, the clustered amide groups with molecular motions restricted in the aggregate state enable these polyamides with luminescence, a phenomenon of AIE. This study provides an approach to pursuing biobased polymers derived from renewable natural products, which combine ultrahigh mechanical strength, excellent elasticity, and strong photoluminescence.

## Methods

### Synthesis of UDA

We revised a procedure reported earlier^11^. UDA was prepared via amidation using 1,3-diamino-propanol (Fig. 1a). A typical procedure is given as follows: 1,3-diamino-propanol (10 mmol, 0.93 g) and methyl 10-undecenoate (20 mmol, 4.13 g) were charged into a 25 mL round-bottom flask. After purging nitrogen at 100 °C for 30 min, the reaction mixture was cooled to 65 °C, and 0.1 mL of sodium methoxide (0.5 mmol, 27 mg in 30wt% methanol) and 3 mL of anhydrous tetrahydrofuran (THF) were added. The reaction ran for 12 h. Pure and white UDA powder (3.0 g, 63% yield) was obtained via recrystallization from methanol twice and dried under vacuum.

### Synthesis of BUDA

Monomer (BUDA) was prepared by esterification between UDA with butyric anhydride (Fig. 1a). A typical procedure is described as follows: UDA (8.0 mmol, 3.38 g), butyric anhydride (8.4 mmol, 1.33 g), 4-dimethylaminopyridine (0.24 mmol, 30.0 mg), and THF (4 mL) were added into a 25 mL round-bottom flask. After reacting at 60 °C for 24 h, deionized water (1 mL) and THF (4 mL) were injected to the mixture to quench the unreacted anhydride. The reaction solution was then poured into dichloromethane and washed with aqueous solutions of NaHCO_3_ and NaCl. White BUDA solid (2.7 g, 69% yield) was obtained by drying the organic phase and evaporating the solvent.

### Preparation of polyamides

Polyamides were prepared via thiol-ene addition polymerization of UDA and BUDA with dithiol. Azobisisobutyronitrile (AIBN) was used as a radical initiator. A typical procedure is described as follows (using BUDA as an example): BUDA (2.0 mmol, 0.99 g), 3,6-dioxa-1,8-octanedithiol (2.0 mmol, 0.38 g), AIBN (0.06 mmol, 10 mg), and THF (2 mL) were charged into a 10 mL flask, purged with nitrogen for 10 min, and reacted at 70 °C for 12 h. The reaction mixture was diluted with THF and precipitated in methanol for several times. The precipitates were dried under vacuum at 40 °C to obtain homopolymer of PBUDA (P0) (64% yield). All other polyamides were prepared in a similar procedure.

### Preparation of polyamide films

P0–P4 films were prepared by solution casting. A typical procedure is described as follows: polyamides (1.0 g) were dissolved in THF (7 mL) and poured into a Teflon mold. Films were dried at room temperature for 2 days, and then under vacuum at 40 °C for 24 h. Fibers of uE4 were prepared via a wire-drawing process. Typically, P4 (1.0 g) was introduced into a flask and heated to 120 °C. After melting, fibers were drawn from the melt using a tweezer.

### Preparation of metal–ligand coordination polyamide films

A typical procedure was described as follows: P4 (700 mg), CuBr (6 wt%, 43.2 mg), and dimethylformamide (6 mL) were added into a 25 mL round-bottom flask. The mixture was purged with nitrogen for 15 min and heated at 65 °C for 8 h, followed by rotary evaporation and vacuum dry at 80 °C for 72 h. Films were obtained by hot press at 120 °C.

### Preparation of uEs

Polymers P0–P4 were performed on a SUNS UTM2502 instrument with a 100 N load cell. The crosshead speed was set at 10 mm min^−1^. The maximum strain was stretched to 100%, 200%, 300%, up to 600% during the first step-cycle tensile deformation. The maximum strain during the second step-cycle tensile deformation was gradually increased from 5 to 40%. The elastic recovery of uEs was calculated using the equation: Elastic recovery = (*ε*_max_ − *ε*_0_)/*ε*_max_, where *ε*_max_ and *ε*_0_, respectively, represent the maximum strain and the strain at 0 MPa in each cycle. The preparation of metal–ligand coordination-based ultra-strong elastomers (uE-Cu) was carried out using the similar procedure to the above.

## Supplementary information

Supplementary Information

## Data Availability

All the data supporting the findings of this study are available within the article and its Supplementary Information file or from the corresponding authors upon request.
