# Supplementary material for: Ultra-strong long-chain polyamide elastomers with programmable supramolecular interactions and oriented crystalline microstructures
Source: Nat Commun. 2019 Mar 21;10:1315. doi: 10.1038/s41467-019-09218-6 (PMC6428834; doi:10.1038/s41467-019-09218-6)
Supplement: Supplementary file 1 — Supplementary Information [file 41467_2019_9218_MOESM1_ESM.pdf]

Supplementary Information

Ultra-strong Long-Chain Polyamide Elastomers with Programmable Supramolecular Interactions and Oriented Crystalline Microstructures

Song et al.

Supplementary Methods

Materials: Methyl 10-undecenoate (TCI, 96%), sodium methoxide (TCI, 30 wt% in methanol), 3,6-dioxa-1,8-octanedithiol (96%, TCI), 1,3-diamino-2-propanol (97%, Macklin), butyric anhydride (98%, Aladdin), and 4-dimethylaminopyridine (99%, Aladdin) were used received. 2,2-Azobis(2-methylpropionitrile) (AIBN) was recrystallized before use. Tetrahydrofuran (99.0%, Sigma-Aldrich) was dried and distilled under vacuum before use. Methanol, dichloromethane, sodium chloride, sodium bicarbonate, and anhydrous magnesium sulfate were supplied by Shanghai Titan Scientific Co., Ltd and used without purification.

Measurements: FT-IR spectra were recorded on a Bruker Tensor 27 FT-IR spectrophotometer. Variable temperature FT-IR (VT-FTIR) experiments were performed on a Bruker Tensor 27 FT-IR spectrophotometer with Eurotherm 2404 temperature controller. The sample was heated from 30°C to 190°C, and the FT-IR spectra were gathered every 10 °C. ^{1}H NMR (600 MHz) and ^{13}C NMR (125 MHz) spectra were recorded on an Agilent DD2 600MHz using $CDCl_3$ as deuterated solvent. Molecular mass and molecular mass distribution of functional polyamides were characterized via Gel Permeation Chromatography (GPC) equipped with an Agilent 1260 HPLC pump, a G1316A refractive index detector, and PL gel column (MIXED-A in the effective molecular weight range of 200-2,000,000 Daltons). Standard polystyrene was used as the calibration polymer with tetrahydrofuran (Aladdin, HPLC grade) as eluent phase at 20 °C and a flow rate of 1 mL min^{-1}. Polymer solutions were filtered over microfilters with a pore size of 0.22 μm before characterization. Differential scanning calorimetry (DSC) curves were obtained using a METZSCH DSC 200 F3 Maia instrument with a nitrogen flow rate of 20 mL/min. Samples were heated from room temperature to 200 °C and cooled to −50 °C at a rate of 10 °C/min. Then samples were heated from −50 °C to 200 °C followed by cooling to −50 °C at a rate of 10 °C/min. Thermal stability was characterized via using METZSCH TG 209 F3 Tarsus thermogravimetric analyzer (TGA) with a nitrogen flow rate of 20 mL min^{-1}. Samples were heated from 20 °C to 100 °C with a heating rate of 30 °C min^{-1}, and kept at 100 °C for 10 min. After cooled to 40 °C, samples were heated to 700 °C with a rate of 10 °C min^{-1}. Tensile tests were performed on SUNS UTM2502 instrument with 100 N load cell at a speed of 10 mm/min. Wide angle X-ray diffraction (WAXD) experiments were conducted using a

SAXSLab Ganesha at the South Carolina SAXS Collaborative. A Xenocs GeniX3D microfocus source was used with a Cu target to generate a monochromic beam with a 0.154 nm wavelength. The instrument was calibrated using National Institute of Standards and Technology (NIST) reference material 640c silicon powder with the peak position at 2θ of 28.44°. A Pilatus 300 K detector (Dectris) was used to collect the two-dimensional (2D) scattering patterns. The 2D images were azimuthally integrated to yield the scattering vector and intensity with SAXSGUI software. The conversion of the resulting intensity versus q data was converted to 2θ using the formula $q=4\pi\lambda^{-1}\sin\theta$, where λ is the X-ray wavelength and 2θ is the total scattering angle. Polarized Optical Microscopy (Olympus BX53) was applied to observe the crystalline morphology. Olympus IX71 fluorescent microscopy was applied to take fluorescent images. Optical Microscopy (Nikon ECLIPSE Ni) was used to measure the diameter of fibers. Fluorescent emission spectra were obtained on Hitachi F-7000 with xenon discharge lamp (150W) as excitation. And UV-vis absorption spectra were obtained on SHIMADZU UV-3600.

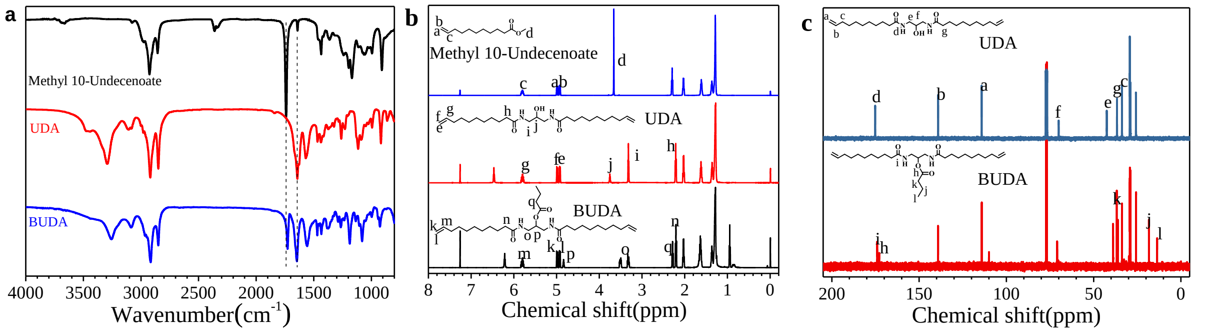

Supplementary Figure 1. (a) FT-IR spectra and (b) ^{1}H NMR spectra of methyl 10-undecenoate, UDA, and BUDA; (c) ^{13}C NMR spectra of UDA and BUDA. $CDCl_3$ was used as deuterated solvent.

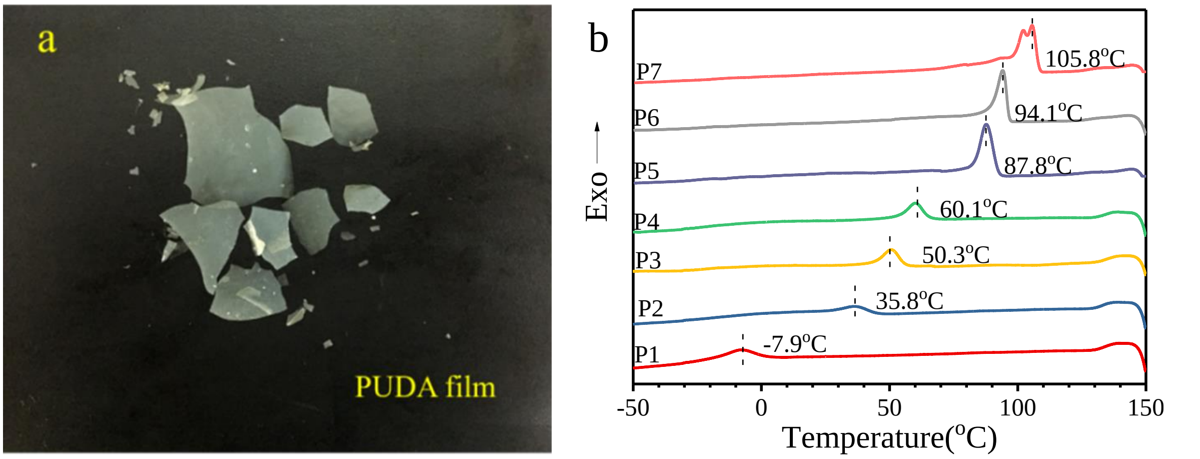

Supplementary Figure 2. (a) Photo of a brittle film of PUDA (P7); (b) DSC cooling curves of P1-P7.

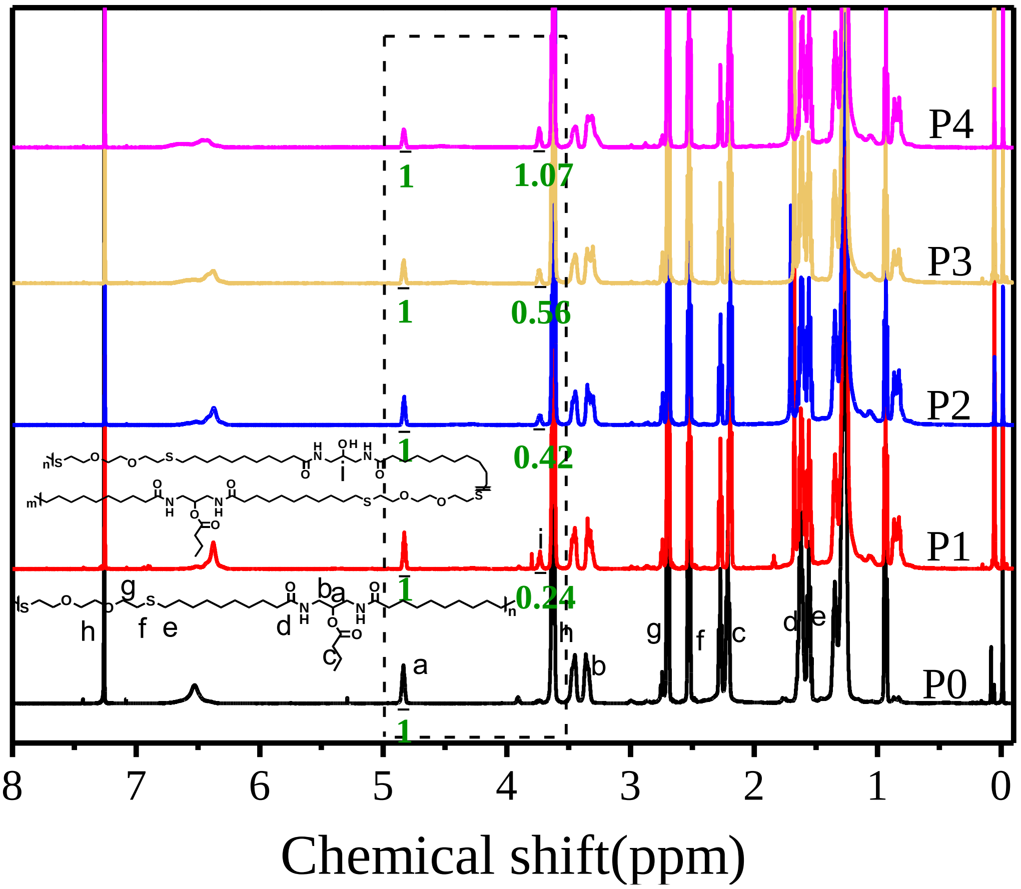

Supplementary Figure 3. ^{1}H NMR spectra of polyamides P0-P4.

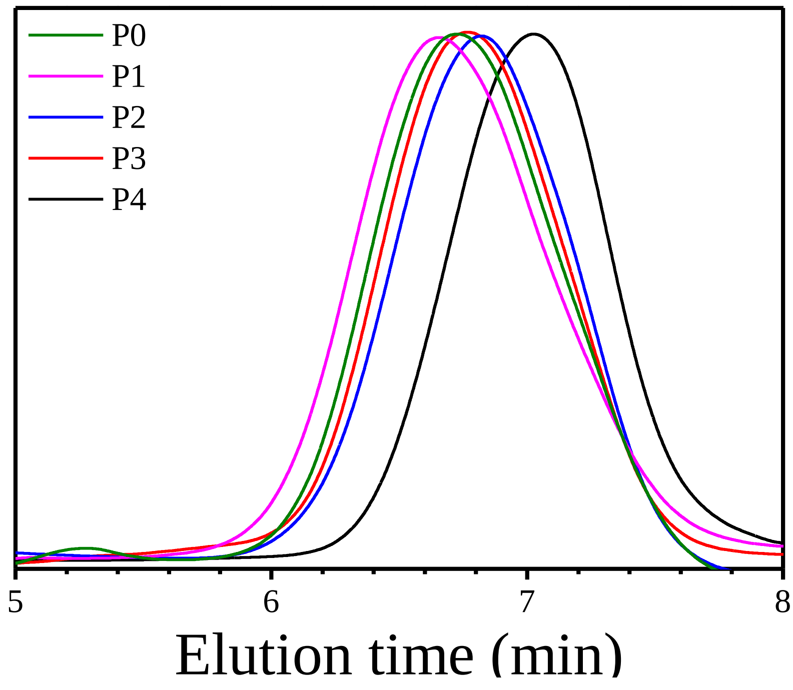

Supplementary Figure 4. GPC curves of P0-P4.

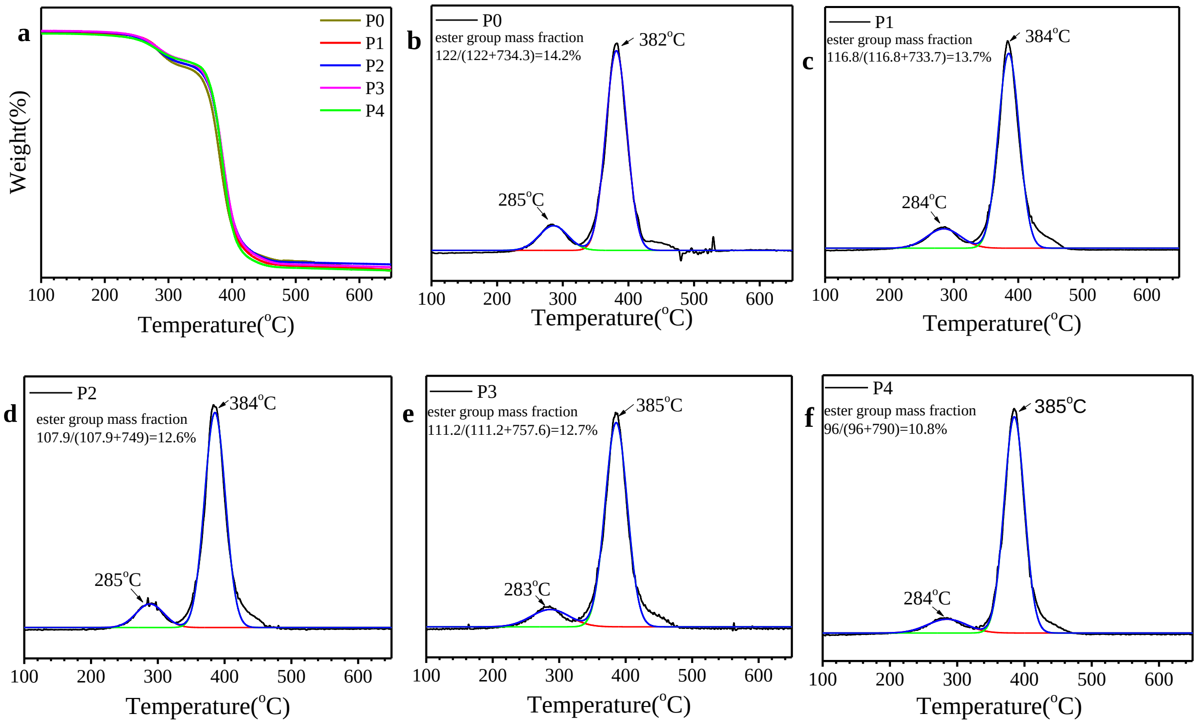

Supplementary Figure 5. (a) Thermogravimetric analysis of P0-P4; (b-f) DTG curves of P0-P4.

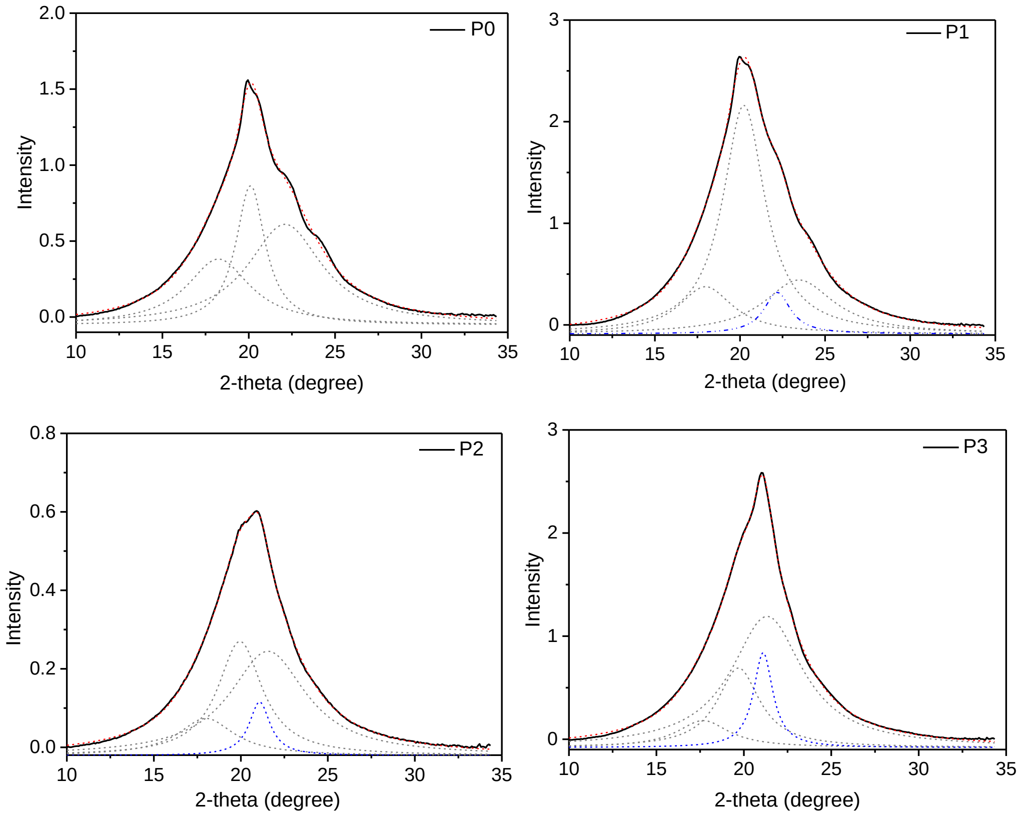

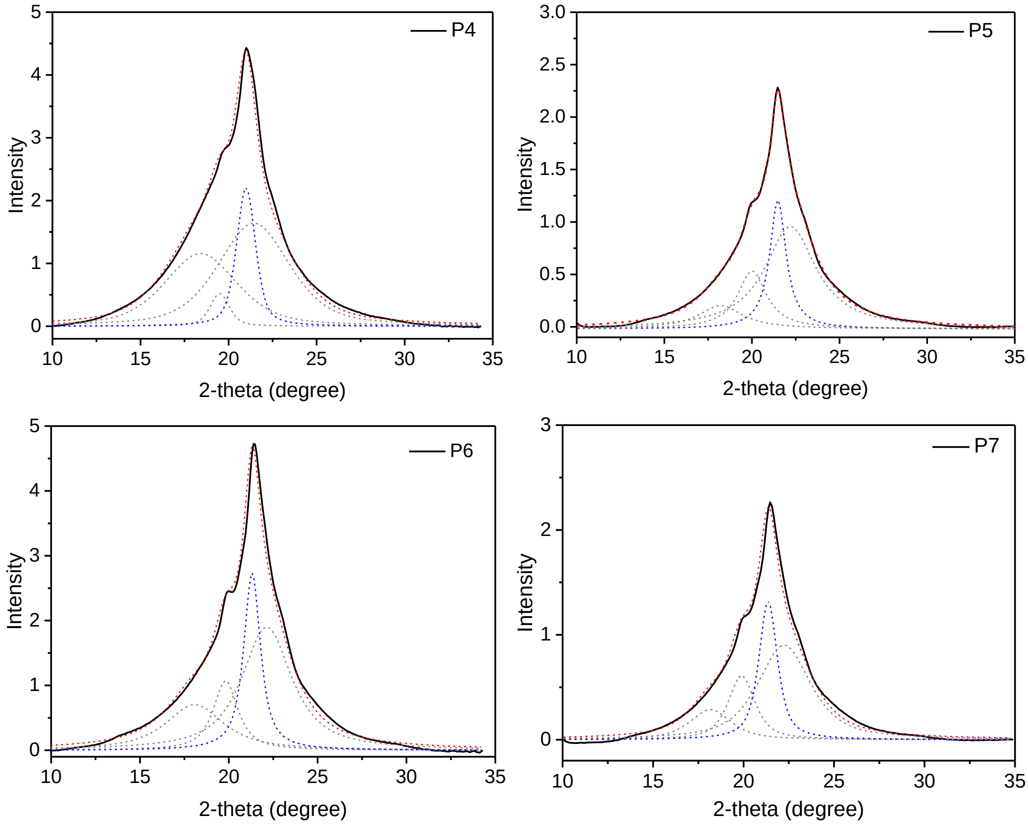

Supplementary Figure 6. WAXD patterns of polyamides (black line: experimental data; red line: fitting sum peak; blue and grey lines: fitting multi-peaks, where blue line was considered as crystalline peak).

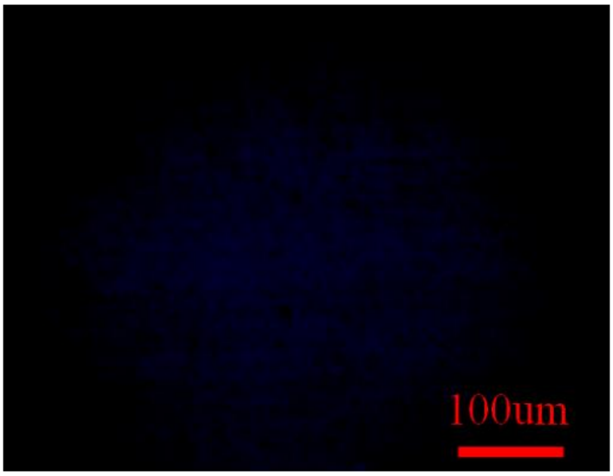

Supplementary Figure 7. POM image of P4, the absence of obvious large crystals indicates the formation of small crystals.

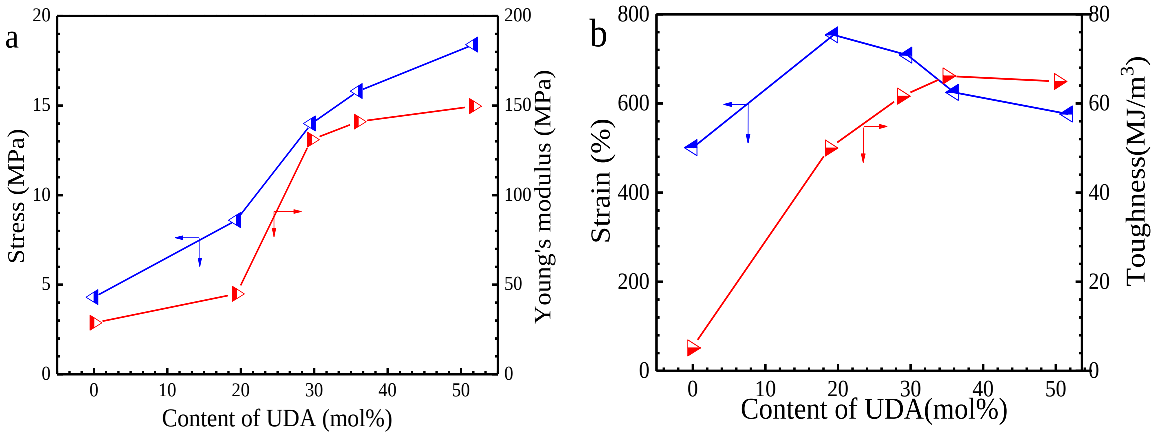

Supplementary Figure 8. The change of (a) ultimate tensile stress and Young's modulus; (b) ultimate tensile strain and toughness as a function of UDA content.

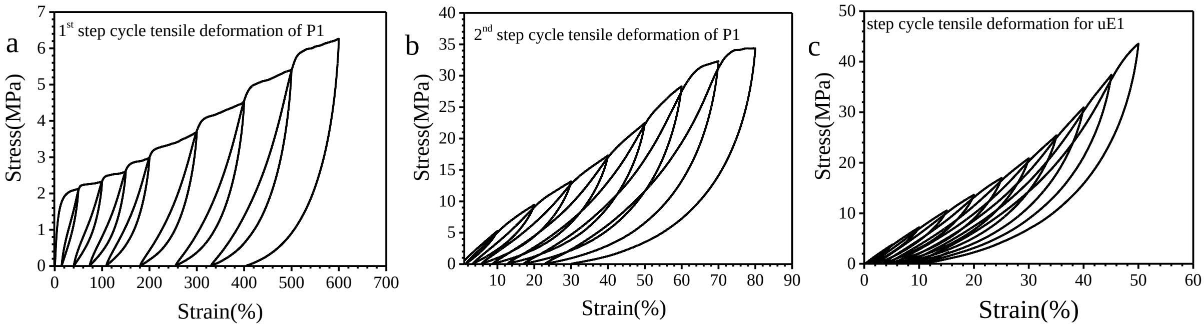

Supplementary Figure 9. Cyclic tensile deformation. (a) 1st step cycle tensile of P1; (b) 2nd step cycle tensile of P1; (c) Stress-strain curves of uE1 during step cycle tensile deformation (this was done after the formation of uE1 with two step-cycle tension of P1).

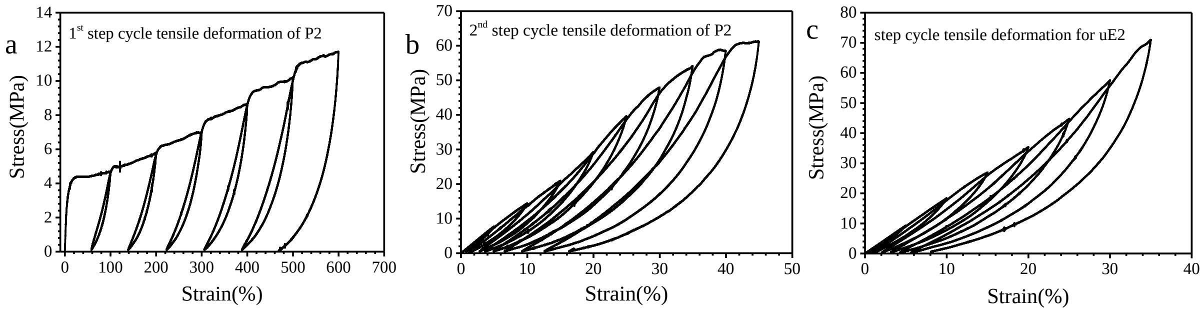

Supplementary Figure 10. Cyclic tensile deformation. (a) 1st step cycle tensile deformation of P2; (b) 2nd step cycle tensile deformation of P2; (c) Stress-strain curves of uE2 during step cycle tensile deformation (this was done after the formation of uE2 with two step-cycle tension of P2).

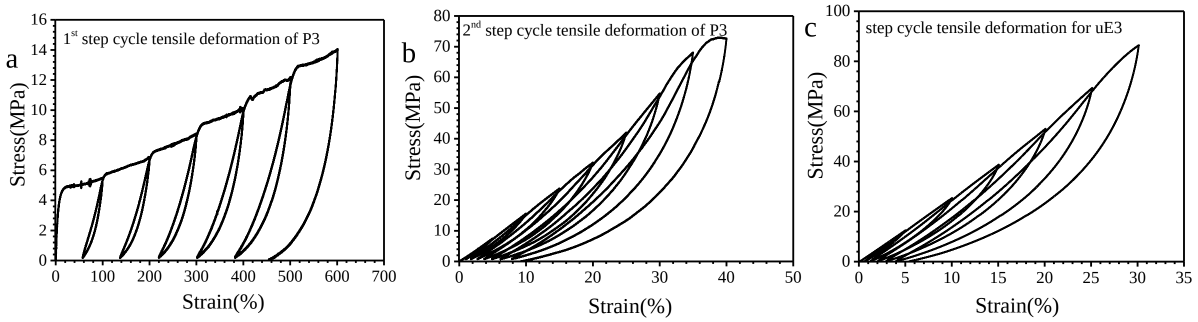

Supplementary Figure 11. Cyclic tensile deformation. (a) 1st step cycle tensile deformation of P3; (b) 2nd step cycle tensile deformation of P3; (c) Stress-strain curves of uE3 during step cycle tensile deformation (this was done after the formation of uE3 with two step-cycle tension of P3).

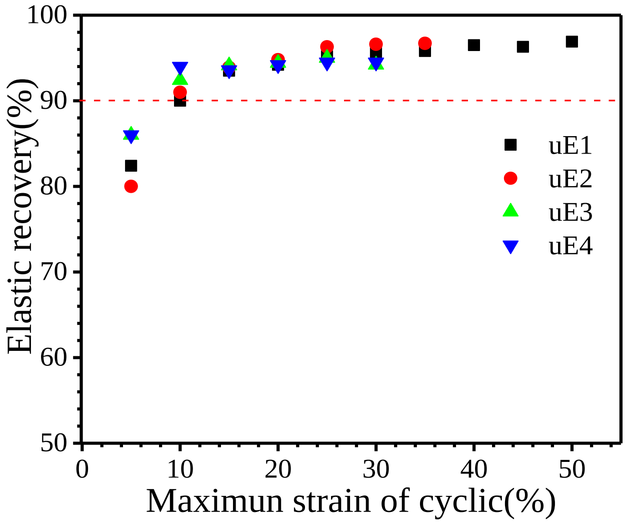

Supplementary Figure 12. The change of elastic recovery of uEs as a function of strain.

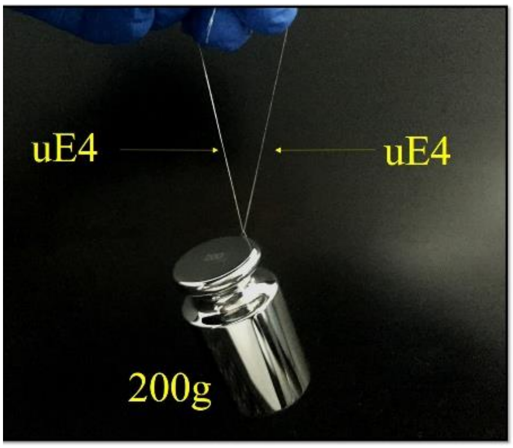

Supplementary Figure 13. A photo shows that 5.9 mg uE4 fiber with a diameter of about 135 μm can easily hold a 200g object.

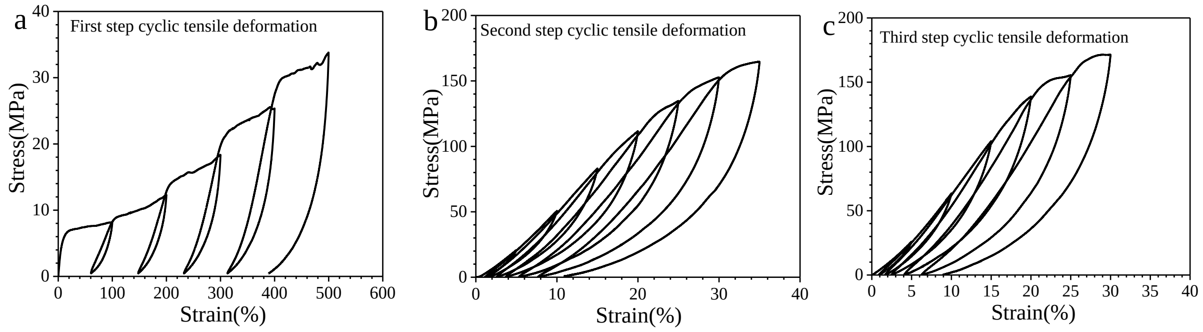

Supplementary Figure 14. Cyclic tensile deformation. (a) 1st step cycle tensile deformation of P4-CuBr (14.4 mol%); (b) 2nd step cycle tensile deformation of P4-CuBr (14.4 mol%); (c) Stress-strain curves of uE4-CuBr (14.4 mol%) during step cycle tensile deformation (this was done after the formation of uE4-CuBr (14.4 mol%) with two step-cycle tension of P4-CuBr (14.4 mol%)).

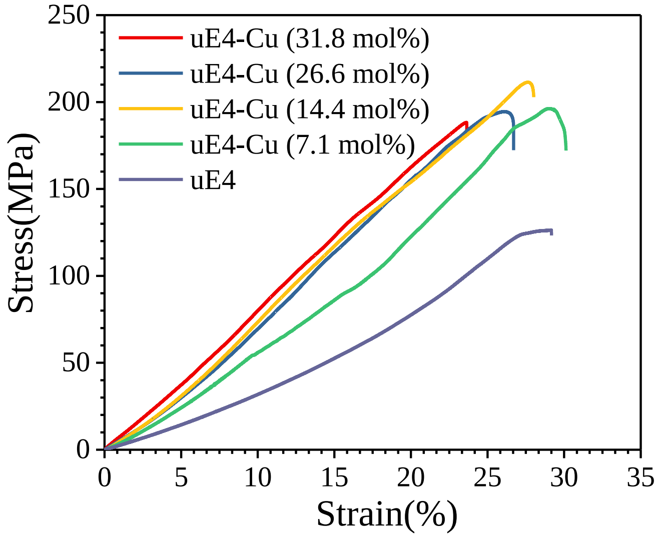

Supplementary Figure 15. Stress-strain curves of uE4-Cu composites with different CuBr contents.

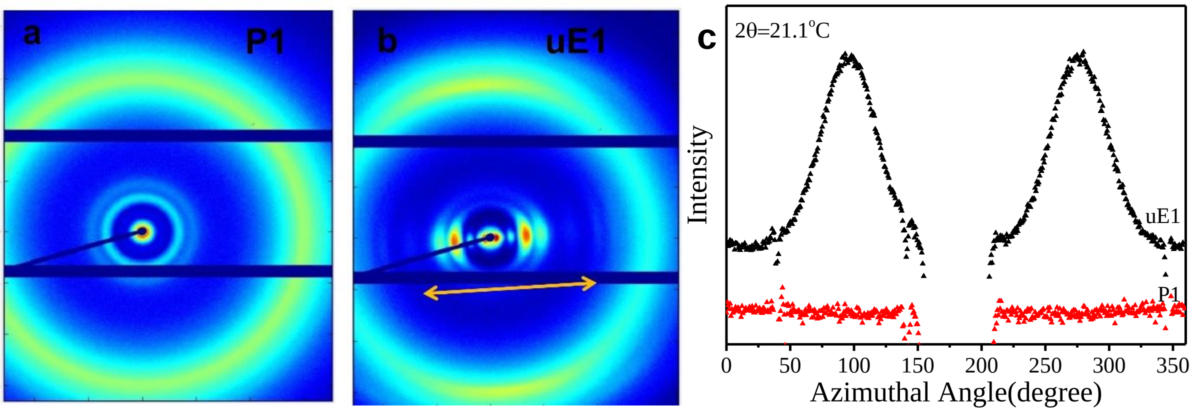

Supplementary Figure 16. Microstructure analysis of uE1: (a) and (b) 2D WAXD patterns of P1 and uE1; (c) WAXD azimuthal intensity profiles for P1 and uE1 at $2\theta = 21.1^{o}$.

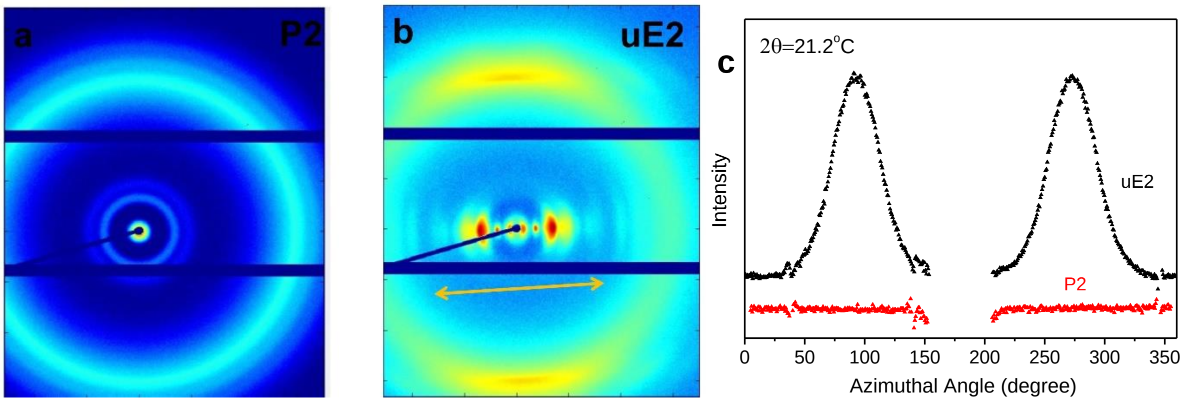

Supplementary Figure 17. Microstructure analysis of uE2. 2D WAXD patterns of (a) P2 and (b) uE2; (c) WAXD azimuthal intensity profiles for P2 and uE2 at $2\theta = 21.2^{o}$.

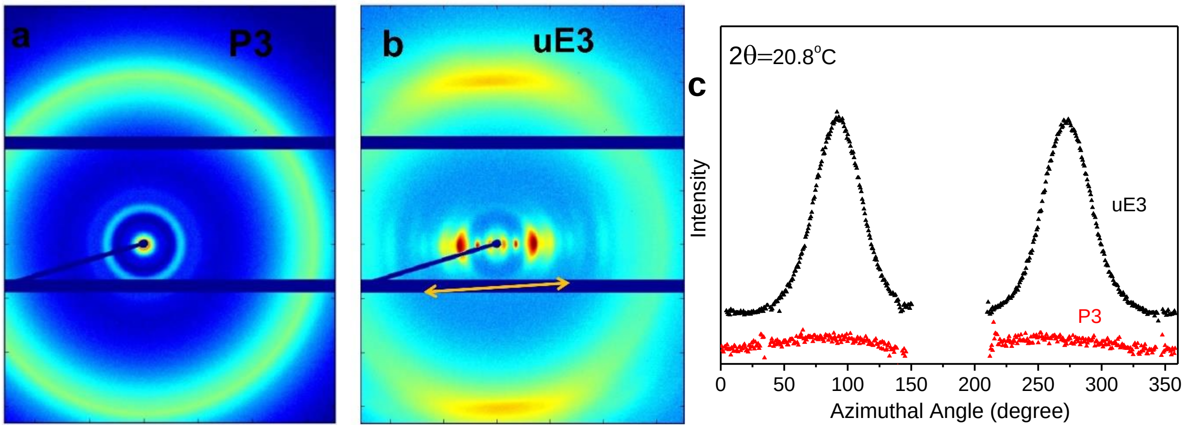

Supplementary Figure 18. Microstructure analysis of uE3. 2D WAXD patterns of (a) P3 and (b) uE3; (c) WAXD azimuthal intensity profiles for P3 and uE3 at $2\theta = 20.8^{o}$.

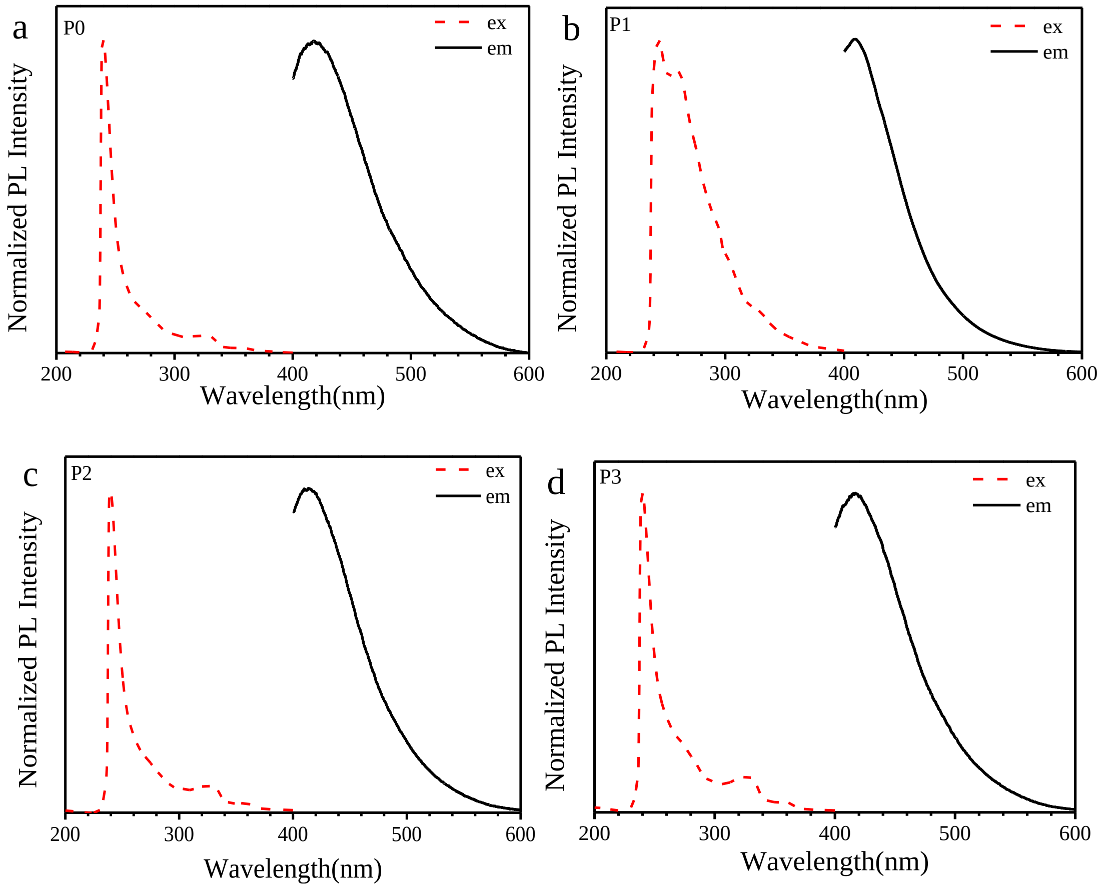

Supplementary Figure 19. UV-vis absorption (red line) and fluorescent emission (black line) spectra of (a) P0; (b) P1; (c) P2; (d) P3.

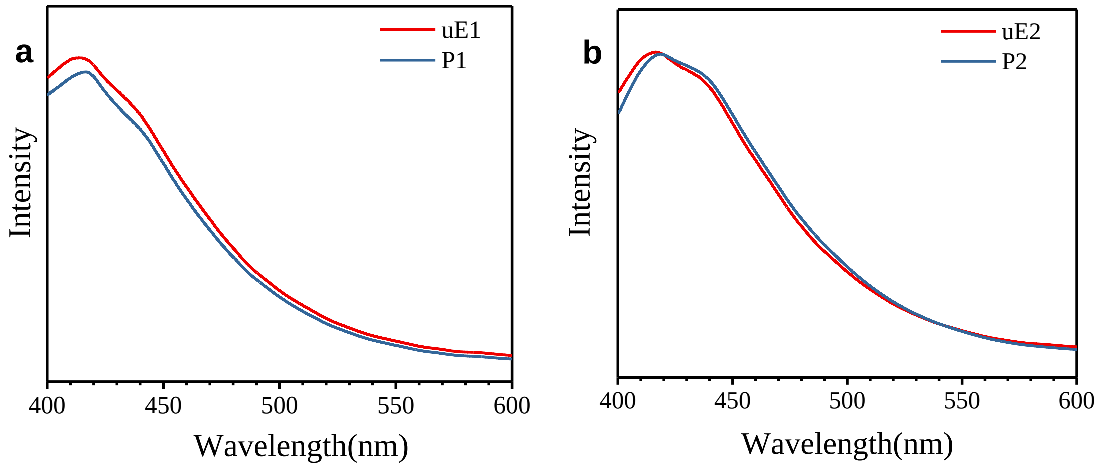

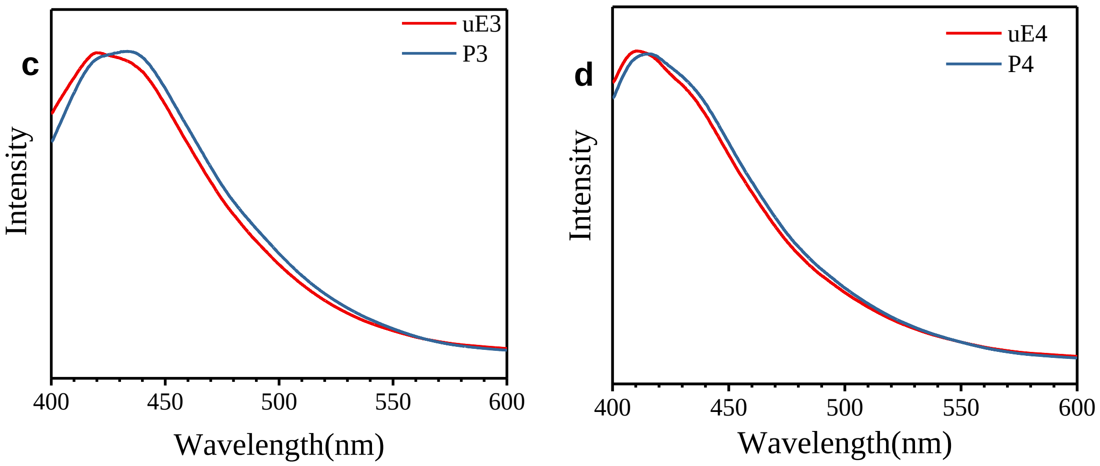

Supplementary Figure 20. Fluorescent emission spectra of uEs (red line) and their corresponding functional polyamides (black line). (a) P1 and uE1; (b) P2 and uE2; (c) P3 and uE3; (d) P4 and uE4.

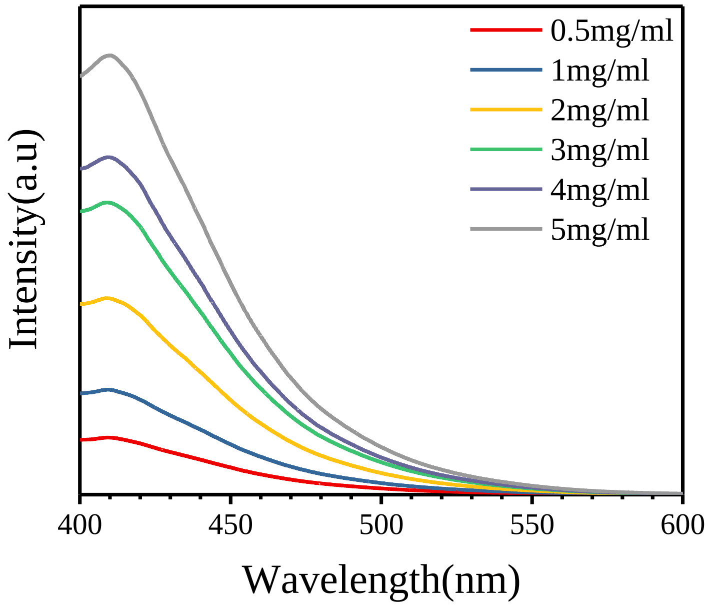

Supplementary Figure 21. Fluorescent emission spectra of P4 with different concentrations.

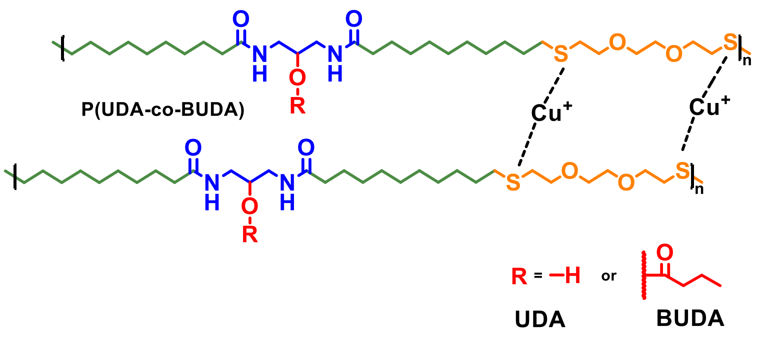

Supplementary Figure 22. Illustration on the formation of cuprous-thioether coordination by adding CuBr into polyamides.

Supplementary Table 1. Characterization of P(UDA-co-BUDA) copolymers.

Sample	UDA feed ratio (mol %)	UDA content (mol%)	M_n (g mol^{-1})	*Đ*	T_g	T_m
**P0**	0	0	34, 100	1.67	-21.6°C	45.3°C
**P1**	10	19.4	37,600	1.72	-21.6°C	49.5°C
**P2**	20	29.6	22,100	1.57	-22.2°C	69.6°C
**P3**	30	36.0	33,500	1.48	-23.2°C	75.6°C
**P4**	40	51.7	20,000	1.56	-23.8°C	80.6°C
**P5**	60	*NT*	*NT*	*NT*	-25.4°C	107.2°C
**P6**	80	*NT*	*NT*	*NT*	-26.6°C	110.1°C
**P7**	100	100	*NT*	*NT*	-29.4°C	122.3°C

NT: NOT Tested due to poor solubility in common organic solvents.

Supplementary Table 2. Crystalline peak position, crystal size and degree of crystallinity (X_c) of P0-P7.

Sample code	UDA content (mol%)	Crystalline peak (2θ)	Crystal size (nm)	X_c (%)
**P0**	0	-	-	0
**P1**	19.4	22.18°	6.74	6.44
**P2**	29.6	21.09°	7.51	6.89
**P3**	36.0	21.12°	7.52	11.4
**P4**	51.7	21.00°	7.21	17.3
**P5**	*N*	21.47°	8.56	20.8
**P6**	*N*	21.35°	7.77	22.8
**P7**	100	21.26°	7.22	25.0

Supplementary Table 3. Mechanical properties of P0-P4.

Sample	Young's modulus (MPa)	ultimate tensile stress (MPa)	ultimate tensile strain (%)	Toughness (MJ M^{-3})
**P0**	33.75 ± 10.1	4.5 ± 1.1	580.4 ± 54.9	5.2 ± 0.9
**P1**	55.6 ±7.7	8.6 ± 1.6	753.8 ± 71.6	50.0 ± 1.3
**P2**	131.0 ± 10.0	14.0 ± 1.5	708.3 ± 50.1	61.6 ± 1.6
**P3**	141.0 ± 7.0	15.8 ± 3.1	624.7 ± 53.2	66.2 ± 1.1
**P4**	149.6 ± 3.1	18.4 ± 2.1	576.3 ± 27.8	64.9 ± 0.7

Supplementary Table 4. Mechanical properties of uEs.

Sample	Stress at break (MPa)	Elongation at break (%)
**uE1**	45.7 ± 1.9	49.6 ± 2.4
**uE2**	72 ± 2.6	37.8 ± 0.7
**uE3**	89.7 ± 13.4	34.1 ± 1.2
**uE4**	115.4 ± 10.9	29.9 ± 3.4

Supplementary Table 5. A comparison of mechanical properties between our polyamides and other long-chain polyamides and polyesters reported in literature.

Polymer	Molecular Weight (kg/mol)	Stress (MPa)	Elongation (%)	Literature
**Long-chain Aliphatic Polyamides**				
Polyamide 6.24	35	33	*N*	Polymer 2000, 41, 3531−3539.
Polyamide 6.34	30	21	*N*	
DAPA Polyamides	16.3 to 43.1	11.9 to 15.5	317 to >600	Polymer 2010, 51, 5895-5902
Nylon-12	17.3	63.7	*N*	Ind. Eng. Chem. 1970, 62, 16−22.
Polyamide 11	14.1	*N*	>100	Eur. Polym. J. 2014, 59, 69–77.
Nylon 6,10	*N*	67	440	Nano Lett 2007, 7, 1178–1185.
Dimer fatty acid polyamides (DAPA)	25	30	400	Eur. Polym. J. 2015, 67, 418–427
Bio-based aliphatic polyamides (BDIS)	*N*	24 to 50	29 to 610	Polymer 2014, 55, 4846-4856
Polyamide 6	21.9	51	27	J. Mater. Chem. 2012, 22, 24081–24091
**P4**	**31.2**	**18.4**	**576**	**This work**
**uE4**	**31.2**	**126.3**	**29.9**	
**uE4-Cu(14.4 mol%)**	***N***	**211.2**	**27.9**	
**Long-chain Aliphatic Polyesters**				
poly(ω-pentadecalactone) (PDL)	64.5	14.5	12	J. Polym. Sci., Part B: Polym. Phys. 2001, 39, 1721−1729.
PDL	45 to 481	13.7 to 20.4	4.5 to 703	Polymer 2010, 51, 1088−1099.
poly(CL-ran-PDL)	62 to 209	9.2 to 17.5	1090 to 1980	Macromolecules 2015, 48, 5845−5854.
Poly(ω-hydroxytetradecanoate)	53 to 140	13.5 to 16.1	5.8 to 729	Biomacromolecules 2011, 12, 3291−3298.
PDL	143 to 305	24.7 to 38.7	900 to 1200	Polym. Chem. 2010, 1, 525−533.
poly(ω-hydroxy fatty ester)s	16 to 440	9.2 to 18.4	1.3 to 5.9	Polym. Int. 2014, 63, 1902−1911.

Polymer	Molecular Weight (kg/mol)	Stress (MPa)	Elongation (%)	Literature
**Long-chain Aliphatic Polyesters** (continued from previous page)				
poly(triacontamethylene triacontanedioate)	*N*	12.5	5	Macromol. Chem. Phys. 1997, 198, 861−869.
poly[1,19-nonadecadiyl-1,19-nonadecanedioate]	30	15.9	619	Green Chem. 2014, 16, 2008−2014.
poly[1,23-tricosadiyl-1,23-tricosanedioate]	39	17.0	678	
TPE-$C_{23}PTMG_{2000}$-65wt%	38	7.7 to 8.2	278 to 326	Polym. Chem. 2015, 6, 7133−7137.
TPE-$C_{23}PPDO_{2000}$-65wt%	19	7.9 to 8.4	718 to 767	

Supplementary References

(1) Yuan L, Wang Z, Trenor NM, Tang C. Robust Amidation Transformation of Plant Oils into Fatty Derivatives for Sustainable Monomers and Polymers. *Macromolecules* **48**, 1320-1328 (2015).

(2) Yuan L, Wang Z, Trenor NM, Tang C. Amidation of Triglycerides by Amino Alcohols and Their Impact on Plant Oil-Derived Polymers. *Polym Chem* **7**, 2790-2798 (2016).

(3) Song L, Wang Z, Lamm ME, Yuan L, Tang C. Supramolecular Polymer Nanocomposites Derived from Plant Oils and Cellulose Nanocrystals. *Macromolecules* **50**, 7475-7483 (2017).
